# Morphological change of a laterally spreading rectal tumor over a short period

**DOI:** 10.1186/1471-230X-13-129

**Published:** 2013-08-19

**Authors:** Hideaki Miyamoto, Yasuhiro Oono, Kuang-l Fu, Hiroaki Ikematsu, Satoshi Fujii, Takashi Kojima, Tomonori Yano, Atsushi Ochiai, Yutaka Sasaki, Kazuhiro Kaneko

**Affiliations:** 1 Department of Gastroenterology, National Cancer Center Hospital East, 6-5-1, Kashiwanoha, Kashiwa City, Chiba 277-8577, Japan; 2 Department of Gastroenterology, Juntendou University Nerima Hospital, Tokyo, Japan; 3 Pathology Division, Research Center for Innovative Oncology, National Cancer Center Hospital East, Chiba, Japan; 4 Department of Gastroenterology and Hepatology, Faculty of Life Science, Kumamoto University, Kumamoto, Japan

## Abstract

**Background:**

Laterally spreading tumors (LSTs) are generally defined as superficial lesions ≥10 mm in diameter that typically extend laterally rather than vertically along the colonic wall. LSTs are usually categorized into 2 subtypes: granular type and nongranular type. Large nodules or depressed areas in granular-type LSTs (LST-Gs) are endoscopic findings of a cancerous component and sometimes represent submucosal invasion. However, the lateral growth and development of LST-Gs remains unclear.

**Case presentation:**

This case report describes a case of 79-year-old woman who underwent total colonoscopy due to a positive fecal occult blood test and was detected a LST-G, about 30 mm in diameter in the lower rectum. The lesion consisted of not only aggregated small and large nodules typically seen in LST-Gs but also the hardly elevated flat parts. In the flat part, there were dilated round pits and no evident capillary vessels. Three months later, the flat part increased in height, the dilated round pits were partly replaced by type IIIL pits, and capillary vessels were evident. The lesion was removed by endoscopic submucosal dissection, and diagnosed pathologically as tubular adenoma. We performed the sequence analyses on *KRAS*, *BRAF*, *NRAS* and *PIK3CA* genes in flat part and nodular part separately, and a mutation of *KRAS* gene at codon 146 was observed at only nodular part, suggesting probable that nodular part be a precancerous lesion.

**Conclusion:**

This is a unique and suggestive case, providing information on progression of LST-Gs at the very early stage to carcinogenesis.

## Background

Laterally spreading tumors (LSTs) are generally defined as superficial lesions ≥10 mm in diameter that typically extend laterally rather than vertically along the colonic wall [1]. Recently, colorectal LSTs have been detected worldwide [2]. LSTs are usually categorized into 2 subtypes: granular type (LST-G) and nongranular type (LST-NG), based on the difference of morphology endoscopically [3]. LST-Gs, particularly of a larger size, are detected more frequently than LST-NGs in the rectum [4]. Large nodules or depressed areas in LST-Gs are endoscopic findings of a cancerous component and sometimes represent submucosal invasion [5]. However, the lateral growth and development of LST-Gs remains unclear. Herein, we present a case involving a rectal LST-G that showed morphological change endoscopically over a short period and a *KRAS* mutation at a rare position (codon 146), which suggested the progression of the LST-G.

## Case presentation

**Figure 1:**
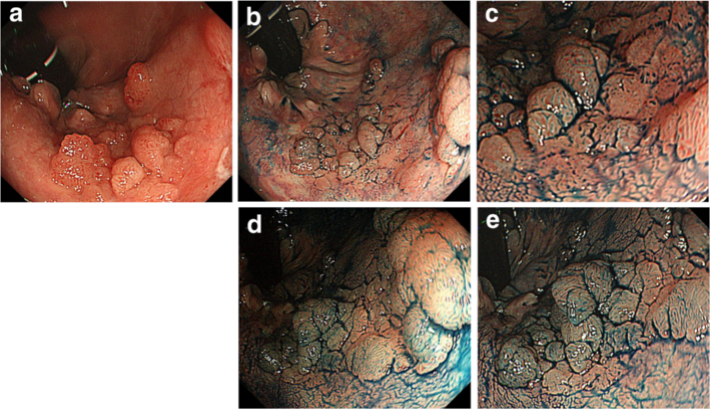
**Colonoscopy revealed a LST-G of approximately 30 mm in diameter in the lower rectum. (a,b)** The lesion consisted of not only aggregated small and large nodules typically seen in LST-Gs, but also hardly elevated flat parts. **(c)** Magnifying chromoendoscopy after 0.4% indigo carmine dye spraying showed a type IIIL pit in the small nodules and a type IV pit in the larger ones, whereas dilated round pits were observed in the hardly elevated flat part. **(d)** Three months later, the nodules that had increased in height and size shows a type IV pit pattern, and a type IIIL pit was seen in the flat area that had increased in height; **(e)** however, the dilated round pits have decreased in number in the flat area. LST-G, granular type laterally spreading tumor.

**Figure 2:**
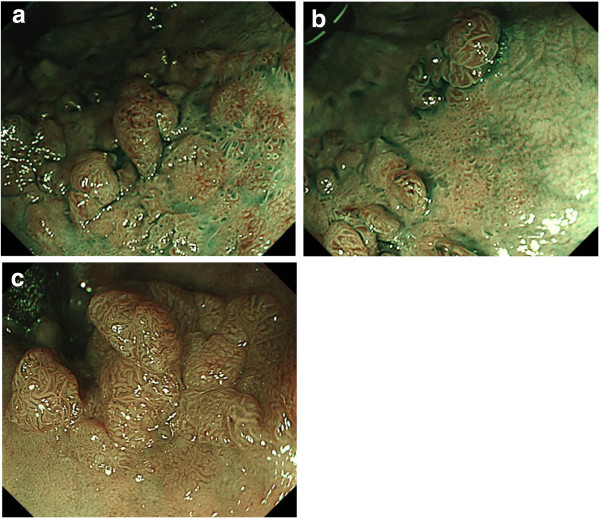
**Narrow-band imaging of the lesion. (a, b)** Magnifying narrow-band imaging revealed MC vessels in the nodular areas, whereas no MC vessel was seen in the flat areas. **(c)** Three months later, the dilated pits in the flat areas have changed into elongated and branched pits, and the MC vessels have become visible. MC vessel, meshed capillary vessel.

A 79-year-old woman underwent total colonoscopy because of a positive fecal occult blood test. Colonoscopy showed a LST-G of approximately 30 mm in diameter in the lower rectum adjacent to the dentate line. Macroscopically, the lesion consisted of not only aggregated small and large nodules typically seen in LST-Gs, but also hardly elevated flat parts (Figure 1a, 1b, 1c). Magnifying chromoendoscopy after 0.4% indigo carmine dye spraying showed a type IIIL pit [6] for small nodules and a type IV pit for the larger ones, whereas dilated round pits were observed in the hardly elevated flat part (Figure 1b, 1c). Magnifying narrow-band imaging (NBI) revealed meshed capillary (MC) vessels (type II capillary pattern according to Sano’s classification [7]) in the nodular areas, whereas no MC vessel (type I capillary pattern according to Sano’s classification) could be seen in the flat areas (Figure 2a, 2b). Based on the above endoscopic findings, this lesion was diagnosed as an adenoma, which is a good candidate for endoscopic local resection.

Approximately 3 months later at the second colonoscopy for endoscopic resection, the nodular parts had increased in height and size, whereas the hardly elevated flat part also increased in height at conventional view. During magnifying chromoendoscopy, the nodules that had increased in height and size showed a type IV pit pattern, and a type IIIL pit was seen in the flat area that had increased in height; however, the dilated round pits decreased in number in the flat area (Figures 1d, 1e, 2c).

**Figure 3:**
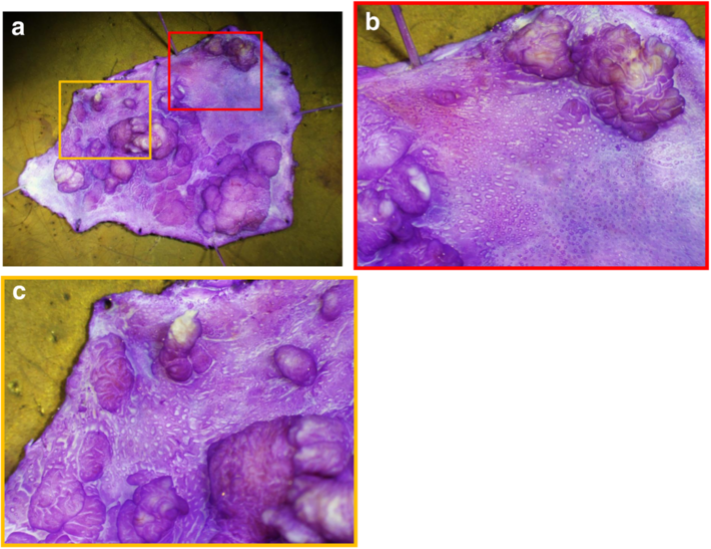
**Stereomicroscopic examination of the resected specimen. (a)** Stereomicroscopic examination showed both nodular parts and flat areas. Moreover, multiple smaller nodules could also be seen within the flat areas. Red frame in 3a: **b**. Yellow frame in 3a: **c**.

**Figure 4:**
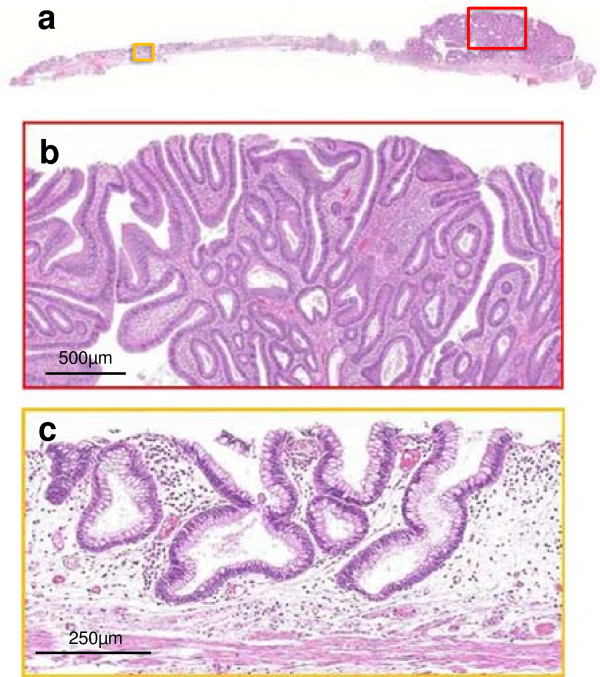
**Histological examination of the resected specimen. (a)** Panoramic view with hematoxylin and eosin staining. **(b)** In the nodular part, there was tubular adenoma with moderate atypia (red frame in 4a). **(c)** In the flat part, there was tubular adenoma with mild atypia (yellow frame in 4a).

The lesion was completely removed en bloc by endoscopic submucosal dissection. Stereomicroscopic examination of the resected specimen showed both nodular parts and flat areas; moreover, multiple smaller nodules were also detected within the flat areas (Figure 3b, 3c). Microscopically, a lower magnified view showed a difference in the density of atypical tubules between the nodular and flat parts (Figure 4a). The nodular part consisted of condensed proliferation of irregular tubules (Figure 4b). In contrast, the flat part consisted of straight tubules that were sparsely distributed compared with nodular part (Figure 4c). Two portions were diagnosed pathologically as tubular adenoma with moderate atypia and tubular adenoma with mild atypia, respectively according to the current pathological criteria.

We performed the sequence analyses on *KRAS* gene at codons 12, 13, 61, and 146, and *BRAF* gene at codon 600, and *NRAS* gene at codons 12, 13, and 61, and *PIK3CA* gene at codons 542, 545, 546, and 1047 to show the significance of detecting the combined nodular and flat lesions like this case. The DNA of nodular or flat lesions were extracted separately from formalin-fixed and paraffin-embedded tissue and analyzed to examine point mutation. The Luminex method was used for *KRAS* gene at codons 61 and 146, and *BRAF* gene at codon 600, and *NRAS* gene at codons 12, 13, and 61, and *PIK3CA* gene at codons 542, 545, 546, and 1047, the Scorpion-ARMS method was used for *KRAS* gene at codons 12 and 13. Interestingly, a mutation of *KRAS* gene at codon 146 was observed at tubular adenoma with moderate atypia of nodular part, however, there was not any mutation examined in tubular adenoma with mild atypia of flat part.

## Discussion

Development and growth of colonic cancers have been analyzed retrospectively from radiographic and endoscopic images [8]. These retrospective studies have demonstrated that early cancers developed slowly, although the speed of their growth accelerated according to the downward invasion of the cancer. According to these retrospective studies, however, only the macroscopic findings such as size and morphological change of polypoid into nonpolypoid were presented. Furthermore, most of the morphological changes were created by deep invasion of the cancers beyond the mucosal layer. It is important to know the growth and development of an adenoma, which is at the very early stage of colorectal neoplasia, according to the concept of the adenoma–carcinoma sequence.

This case is unique and suggestive because the serial endoscopic pictures provided not only configurational changes of enlarged nodules and increased height of flat lesions but also detailed changes of pit patterns and capillary patterns, which supported the macroscopic changes. Magnifying image-enhanced endoscopy (IEE) shows both pit patterns and capillary patterns for predicting histology in vivo without endoscopic biopsies. Therefore, magnifying IEE enables distinction of non-neoplastic and neoplastic lesions in addition to selection of good candidates for endoscopic local resection. In this case, the nodules increased in size and height macroscopically. The small nodule was composed of a type IIIL pit pattern at first colonoscopy, and a type IV pit pattern was seen in the enlarged nodule. In general, the type IV pit represents a longer gland than that associated with the type IIIL pit pattern [6], which is consistent with the macroscopic changes of the nodules that increased in size and height.

The flat part showed type IIIL and dilated round pits. At first, because magnifying NBI revealed no evident capillary vessel around the dilated round pit, we diagnosed it as a non-neoplastic lesion. At the second colonoscopy, the dilated round pits were partly replaced by type IIIL pits, and capillary vessels were evident on magnifying NBI, which suggested that the flat part was neoplastic. Considering that the dilated round pit, which was histologically consistent with tubular adenoma, was observed in the flat part of the resected specimen under stereomicroscopic observation and was histologically consistent with tubular adenoma, the dilated pit may have been the precursor of a type IIIL pit.

Furthermore, it was difficult to identify the margin of this lesion exactly, because there is little difference in height between the flat part and surrounding normal mucosa, and magnifying IEE showed non-neoplastic images. Therefore, pathological analyses in detail are needed to explore the key findings reflecting characteristic endoscopic image like this case. Poor recognition for lesion like this would result in incomplete endoscopic resection, and potentially be involved in the local recurrence. It is important to examine colonoscopy with indigo carmine dye spraying carefully in order to diagnose demarcation of the lesion exactly and to achieve a complete resection.

*KRAS* is one of the most investigated genes in colorectal neoplasia, and mutations of the gene have been observed in 30–40% of sporadic colon neoplasia [9,10]. *KRAS* mutations are thought to be related to the conversion of low-grade adenomas to high-grade adenomas, and also to protrusive growth [11,12]. Interestingly, a mutation of *KRAS* gene at codon 146 was observed in only nodular part in this case. Recent studies have shown the frequency of codon 146 mutation ranging from 1.0% to 4.0% in CRCs [13-15], however, *KRAS* mutation at codon 146 is related to resistance to cetuximab plus irinotecan and is recognized to be an important mutation of *KRAS* gene [13]. There have been few studies on the relationship between *KRAS* mutation at codon 146 and LST. Several questions are raised now, such as whether flat area is a precancerous lesion of LST-G, whether this type of lesion already has a mutation in tubular adenoma with moderate atypia, and whether this finding is a trigger leading to carcinoma in short duration. Further analyses are necessary to respond these questions using a large scale of this type lesion.

## Conclusions

This case is unique and suggestive because it provided information on the progression of an LST-G at the very early stage. Detailed image analysis of pit patterns and capillary patterns obtained by magnifying IEE was useful to support understanding the macroscopic morphological changes. And a *KRAS* mutation at codon 146 was observed in not flat part but only nodular part, therefore in this case, suggesting that a *KRAS* mutation might be associated with morphological change. Further molecular study of the dilated round pit seen in this case is needed to elucidate the carcinogenesis of LST-G.

## Consent

Written informed consent was obtained from the patient to mutational analyses and publication of this Case report. A copy of the written consent is available for review by the Editor of this journal.

## Abbreviations

LST: Laterally spreading tumor; LST-Gs: Granular-type LSTs; NBI: Narrow-band imaging; MC: Vessels, meshed capillary vessels; IEE: Image-enhanced endoscopy.

## Competing interests

The authors declare that they have no competing interests.

## Authors’ contributions

HM was responsible for the design and drafting of the manuscript. YO, KF, HI, SF, TK, TY and SY were responsible for the conception and revision of the manuscript. SF was responsible for the pathological diagnosis. HM, YO and SF were responsible for sequence data interpretation. SF and AO were responsible for material support for mutation analyses. KK was responsible for the final review and revision of the manuscript and the supervision of the study. All authors read and approved the final manuscript form.

## Pre-publication history

The pre-publication history for this paper can be accessed here:

http://www.biomedcentral.com/1471-230X/13/129/prepub
